# C-955-10. Wound Excision Drives Hypermetabolism and Cachexia in Mice with Scald Burn Injuries

**DOI:** 10.1093/jbcr/irag033.159

**Published:** 2026-04-06

**Authors:** Jaycelyn Hall, Mary Barre, Lillie D Treas, Abby Barlow, Meagan S Kingren, Esther H Teo, Craig Porter

**Affiliations:** University of Arkansas for Medical Sciences, Little Rock, AR; Arkansas Children's Research Institute, Little Rock, AR; Arkansas Children's Research Institute, Little Rock, AR; Arkansas Children's Research Institute, Little Rock, AR; University of Arkansas for Medical Sciences, Little Rock, AR; University of Arkansas for Medical Sciences, Little Rock, AR; University of Arkansas for Medical Sciences, Little Rock, AR

## Abstract

**Introduction:**

Increased energy expenditure (hypermetabolism) is a hallmark of the stress response to burns. While mice are commonly used to model burn-induced hypermetabolism, emerging evidence suggest that burn injury alone may not result in significant acute hypermetabolism in mice. Here, we tested the hypothesis that burn wound excision is required to fully model burn-induced hypermetabolism in mice.

**Methods:**

Six week old C57BL/6 J mice (n = 12-18 per group; 50% male) were acclimated to thermoneutral housing (30°C) for six weeks. All mice underwent a baseline body composition scan. Thereafter, mice were randomized to undergo either a sham (control), scald burn (burn), wound excision (excision) or scald burn followed by wound excision (burn+excision) procedure while under anesthesia. Burns and wound excisions involved ~15% of the total body surface area. All mice then recovered for 14-days while individually housed in metabolic phenotyping cages that allowed water vapor loss, resting energy expenditure (REE), and total energy expenditure (TEE) to be continuously measured. At day 14 post injury, mice underwent a second body composition scan prior to euthanasia.

**Results:**

There were no fatalities post injury in the control or burn group, whereas 22% (n = 4) of the excision group and 33% (n = 6) of the burn+excision group died or were euthanized prior to the end of the 14-day recovery period. Relative to the control (6.3 ± 0.2 kcal/day), there was an acute increase in TEE in burn (7.4 ± 0.2 kcal/day), excision (10.1 ± 0.2 kcal/day), and burn+excision groups (10.8 ± 0.3 kcal/day) (*p*<.001 for all). Increased TEE was driven by increased REE in burn (0.24 ± 0.01 kcal/hr.), excision (0.36 ± 0.02 kcal/hr), and burn+excision groups (0.37 ± 0.01 kcal/hr.) when compared to control mice (0.16 ± 0.01 kcal/hr) (*p*<.001 for all), where REE was 153 ± 6%, 223 ± 21% and 237 ± 13% above that of control in burn, excision and burn+excision, respectively (*p*<.001 for all). Compared to control, wound excision (alone or combined with burns) resulted in greater loss in body mass (*p*<.01), bone mineral content (*p*<.01), fat mass (*p*<.001), and fat free mass (*p*<.001) than burn injury alone. This cachectic response to excision was more pronounced in male mice compared to female mice.

**Conclusions:**

Wound excision is a key driver of the acute hypermetabolic catabolic stress response to burns in mice.

**Applicability of Research to Practice:**

Rodent models should incorporate burn wound excision to more accurately model the pathophysiological stress response to burns.

**Funding for the study:**

NIGMS.

